# Reemergence of *Brucella melitensis* Infection in Wildlife, France

**DOI:** 10.3201/eid2009.131517

**Published:** 2014-09

**Authors:** Bruno Garin-Bastuji, Jean Hars, Antoine Drapeau, Moulay-Ali Cherfa, Yvette Game, Jean-Marie Le Horgne, Séverine Rautureau, Eric Maucci, Jean-Jacques Pasquier, Maryne Jay, Virginie Mick

**Affiliations:** French Agency for Food, Environmental, and Occupational Health and Safety, Maisons-Alfort, France (B. Garin-Bastuji, A. Drapeau, M.-A. Cherfa, M. Jay, V. Mick);; French Hunting and Wildlife Agency, Gières, France (J. Hars);; Departmental Veterinary Laboratory of Savoie, Chambery, France (Y. Game);; Departmental Veterinary Services of Savoie, Chambery (J.-M. Le Horgne);; French Ministry of Agriculture, Agro-Food Industry, and Forest, Paris, France (S. Rautureau);; Departmental Veterinary Laboratory of Haute-Savoie, Seynod, France (E. Maucci);; Departmental Hunters Association of Haute-Savoie, Villy-le-Pelloux, France (J.-J. Pasquier)

**Keywords:** brucellosis, Brucella melitensis, bacteria, alpine ibex, chamois, massif, wildlife, ruminants, reservoir, France

**To the Editor:** Brucellosis is a worldwide zoonosis caused by *Brucella* spp. France has been free of bovine, ovine, and caprine brucellosis (caused by *B*. *abortus* or *B*. *melitensis*) since 2003 (*1*). In early 2012, an outbreak of bovine and human brucellosis caused by *B*. *melitensis* biovar 3 (*Bmel*3) occurred in a French Alp massif (mountainous region), where the last reported outbreak occurred in 1999 (Technical Appendix Figure) (*2*). This outbreak suggested the persistence or reemergence of *Brucella* spp. in livestock.

An extensive investigation was conducted that involved 40 animal herds with direct links to the outbreak. Six months later, blood samples from each adult animal in any herd (12,116 animals in 205 herds) that grazed during the summer of 2012 in the massif underwent serologic analysis. However, no other case was identified in this population (Technical Appendix Table 1). Therefore, a potential wildlife source was investigated.

Wild ruminants in the study area were the following species: hunted red deer (*Cervus elaphus*), roe deer (*Capreolus capreolus*), chamois (*Rupicapra rupicapra*), and protected alpine ibex (*Capra ibex*). Although *B*. *abortus* and *B*. *suis* infections have been reported in numerous wildlife species (*3*), *B. melitensis* has rarely been isolated from wildlife, and only sporadic cases of infection have been reported in Europe, in chamois and alpine ibex in the Alps (*4**,**5*) and in Iberian ibex (*Capra pyrenaica hispanica*) in the Pyrenees (*6*). These cases were considered to be caused by spillover from domestic ruminants, which suggests that these wild species are unable to sustain the infection (*3*).

We conducted our investigation during the fall–winter of 2012–2013 in the entire massif where the outbreak occurred. Blood, lung, spleen, and testes or uterus samples were obtained from all hunted animals. French Authorities authorized the killing of 12 seropositive or diseased alpine ibex with clinical signs of brucellosis (i.e., arthritis or orchitis) among 30 captured animals.

All serum samples were tested according to standards of the World Organisation for Animal Health (Paris, France) for diagnosis of brucellosis in small ruminants by using by the Rose Bengal test (RBT) and the complement fixation test (CFT) (*7*), and by indirect ELISA (IDEXX, Montpellier, France) and competitive ELISA (cELISA; Ingenasa, Madrid, Spain). When blood samples were unsuitable for RBT or CFT or were missing, a lung extract was tested by only the 2 ELISAs. Culture was only performed on samples from seropositive animals (Technical Appendix Table 1) (*8*). A *Brucella* genus–specific real-time PCR was also used (*9*).

A total of 129 hunted ruminants (55 chamois, 30 red deer, 44 roe deer) were tested. No clinical signs were observed, except for arthritis in the knee of 1 chamois. All ruminants were seronegative except for the chamois, which showed positive results in the RBT, CFT, and cELISA, and 1 red deer, which showed a weakly positive result in the cELISA, but negative results by culture and real-time reverse transcription PCR. *Bmel*3 was isolated from the chamois (Appendix Table 1).

Among 289 alpine ibex observed in the massif, 24 were killed (22 randomly sampled animals that showed 2 diagnostic lesions at necropsy [arthritis in the knee and mammary abscesses]) and 2 diseased animals (arthritis in the knee and orchitis), and samples from these animals were subjected to serologic analysis. Ten alpine ibex (including the 2 diseased animals) showed positive results in the RBT, CFT and both ELISAs, and 2 showed positive results only for both ELISAs. Thus, the prevalence of *B. melitensis* in randomly captured animals was 45% (10/22; 95% CI 24.6%–66.3%) (Technical Appendix Table 1).

*Bmel*3 was isolated from 5 of 11 seropositive alpine ibex (1 alpine ibex was killed in an avalanche) and from 3 seropositive but culture-negative ibex, which also showed positive results by PCR (Technical Appendix Table 2). Multilocus variable number tandem repeat analysis showed similarity among all strains isolated in this study and strains isolated from local domestic outbreaks >13 years ago (*10*).

Although persistence of *B*. *melitensis* in wild ruminants has not been reported, and these animals are considered an epidemiologic dead-end reservoir (*3*), the unexpected prevalence observed (≈50%) suggests that alpine ibex could be the source of bovine brucellosis reemergence in the study area in France. Strict surveillance policies have prevented infection of domestic livestock with *B. melitensis* in the study area since 1999. However, cohabitation of domestic and wild ruminants on pastures during the summer is rare but possible. Clinical signs and lesions observed in chamois and alpine ibex are consistent with those reported for chamois and alpine ibex with brucellosis (*4**,**5*). Positive cultures were obtained from organ samples (knee, testes, and lymph nodes) but also from urogenital fluids, which indicates the potential for excretion of the organism.

The fact that births occur during periods and in places where female alpine ibex are not in close contact with other wild/domestic species (because of higher altitude or rocky peaks) could explain the low transmission rate of *B. melitensis* to these animals. It also suggests that the venereal route might contribute to the transmission within alpine ibex during the mating season in winter. This report demonstrates the need for maintaining an active/reactive surveillance system for livestock and wildlife in brucellosis-free regions.

Technical Appendix Test results (bacteriologic and PCR) for domestic and wild ruminants and areas in France in which alpine ibex and chamois were found infected with *Brucella melitensis*.
