# Supplementary material for: Reemergence of Brucella melitensis Infection in Wildlife, France
Source: Emerg Infect Dis. 2014 Sep;20(9):1570–1. doi: 10.3201/eid2009.131517 (PMC4178400; doi:10.3201/eid2009.131517)
Supplement: Technical Appendix — Test results (bacteriologic and PCR) for domestic and wild ruminants and areas in France in which alpine ibex and chamois were found infected with Brucella melitensis. [file 13-1517-Techapp-s1.pdf]

Article DOI: http://dx.doi.org/10.3201/eid2009.131517

Reemergence of *Brucella melitensis* in Wildlife, France

Technical Appendix

Technical Appendix Table 1. Test results for domestic and wild ruminants for infection with *Brucella melitensis*, France*

			Serologic tests														Bacteriologic and PCR	
							iELISA‡				cELISA§							
	No.	No.	RBT		CFT†		Serum		Lung¶		Serum		Lung¶		Total		Culture**	RT-PCR††
Animal	herds	animals	+/–	NR	+/–	NR	+/–	NR	+/–	NR	+/–	NR	+P/–	NR	positive#		+/–	+/–
Domestic																		
Cattle	160	8,489	31/8,453	5	11/31	0	ND[j]	ND	ND	ND	ND	ND	ND	ND	11		0/11	ND
Sheep and goats	45	3,634	5/3,627	2	3/5	0	ND	ND	ND	ND	ND	ND	ND	ND	3		0/3	ND
Wild																		
Roe deer‡‡§§	NA	44¶¶	0/20	24	0/13	31	0/26	18	0/16	2	0/26	18	0/16	2	0		ND	ND
Red deer‡‡§§	NA	30	0/24	6	0/18	12	0/28	2	0/2	0	1/27	2	0/2	0	1		0/1	0/1
Chamois‡‡§§	NA	55	1/33	21	1/29	25	0/44	11	0/11	0	1/43	11	0/11	0	1		1/0	ND
Alpine ibex	NA	24##	12/12	0	10/6	8	12/12	0	NR	NR	12/12	0	NR	NR	12		5/6	3/3

*RBT, Rose Bengal test; CFT, complement fixation test; iELISA, indirect ELISA; cELISA, competitive ELISA; RT-PCR, real-time PCR; +/–, no. positive/no. negative; NR, no result (samples were missing or unsuitable for testing because of poor quality or inconclusive results [hemolysis or anti-complementary activity]); ND, not determined.
†CFT – cutoff value = 20 international CFT units/mL.
‡iELISA cutoff value 110%–120%.
§cELISA: cutoff value = 40%.
¶Lung extract was tested by ELISAs only when corresponding serum was missing.
#Number of domestic animals with a positive result by RBT and CFT, number of wild animals with a positive result by either test.
**Culture was performed only for seropositive animals: on retropharyngeal, retromammary, and iliac lymph nodes from domestic animals; on spleen and testes or uterus from hunted animals; and on retropharyngeal, retromammary, and superficial inguinal or iliac lymph nodes, uterus or testes, urine (directly sampled from the bladder), vaginal or preputial swab specimens, udder; and fluid from arthritic area from necropsied alpine ibex.
††RT-PCR was performed for seropositive/culture-negative animals, on testes and spleen from red deer, and on lymph nodes from alpine ibex.
‡‡Total number of animals sampled was 88% (roe deer), 60% (red deer), and 110% (chamois) of the target populations to detect at least 5% prevalence at a 95% CI.
§§Age: chamois, 6 mo–11 y; red and roe deer, young, subadults, and adults.
¶¶44 animals were hunted but blood or lung extract from only 42 animals showed a reliable result.
##Including 2 males with clinical signs and 22 animals randomly captured.

Technical Appendix Table 2. Bacteriologic and PCR results for wild animals seropositive for infection with *Brucella melitensis*, France*

				Serologic tests†				Bacteriologic and PCR	
Animal	ID no.	Sex	Age, y	RBT	CFT	iELISA‡	cELISA§	Culture	PCR
Red deer	4932	M	Adult	–	–	– (57.15)	+ (54.64)	–¶	–¶
Chamois	7464	F	7	+	+ (213)	– (21.93)	+ (94.51)	+#	ND
Alpine ibex	83	M	11	+	+ (80)	+ (306.97)	+ (94.23)	+**	ND
	84	M	13	+	+ (160)	+ (334.80)	+ (95.46)	+††	ND
	2	F	9	+	+ (53)	+ (219.17)	+ (95.31)	–	–
	3	F	12	+	+ (40)	+ (215.97)	+ (86.72)	–	–
	4	F	11	+	+ (320)	+ (251.58)	+ (95.98)	–	+‡‡
	5	F	12	–	–	+ (234.19)	+ (82.98)	–	+§§
	13	F	9	+	+ (320)	+ (288.08)	+ (96.30)	+¶¶	ND
	15	F	14	+	+ (320)	+ (336.27)	+ (94.61)	+##	ND
	16	F	9	+	+ (160)	+ (266.81)	+ (95.72)	–	–
	24	M	11	–	–	+ (340.37)	+ (93.04)	ND***	ND***
	25	F	13	+	+ (93)	+ (248.32)	+ (93.89)	–	+§§
	26	F	8	+	+ (426)	+ (303.06)	+ (96.23)	+†††	ND

*ID, identification; RBT, Rose Bengal test; CFT, complement fixation test; iELISA, indirect ELISA; cELISA, competitive ELISA; –, negative; +, positive; ND, not determined.
†Values are international CFT units/mL (CFT – cutoff value = 20 international CFT units/mL) or percentages (ELISAs).
‡iELISA cutoff value was 110%–120%.
§cELISA cutoff value was 40%.
¶Spleen/testes.
#Arthritis fluid/testes.
**Arthritis fluid, spleen, preputial swab, urine, right testicle, retropharyngeal specimen, lymph nodes.
††Arthritis fluid, nuchal abscess, spleen, preputial swab, urine (directly sampled from the bladder), right testicle, retropharyngeal specimen, lymph nodes.
‡‡Retropharyngeal specimen, lymph nodes.
§§Iliac region, lymph nodes.
¶¶Arthritis fluids.
##Udder, mammary abscess.
***Died in avalanche.
†††Vaginal swab, uterus, retropharyngeal specimen, lymph nodes.

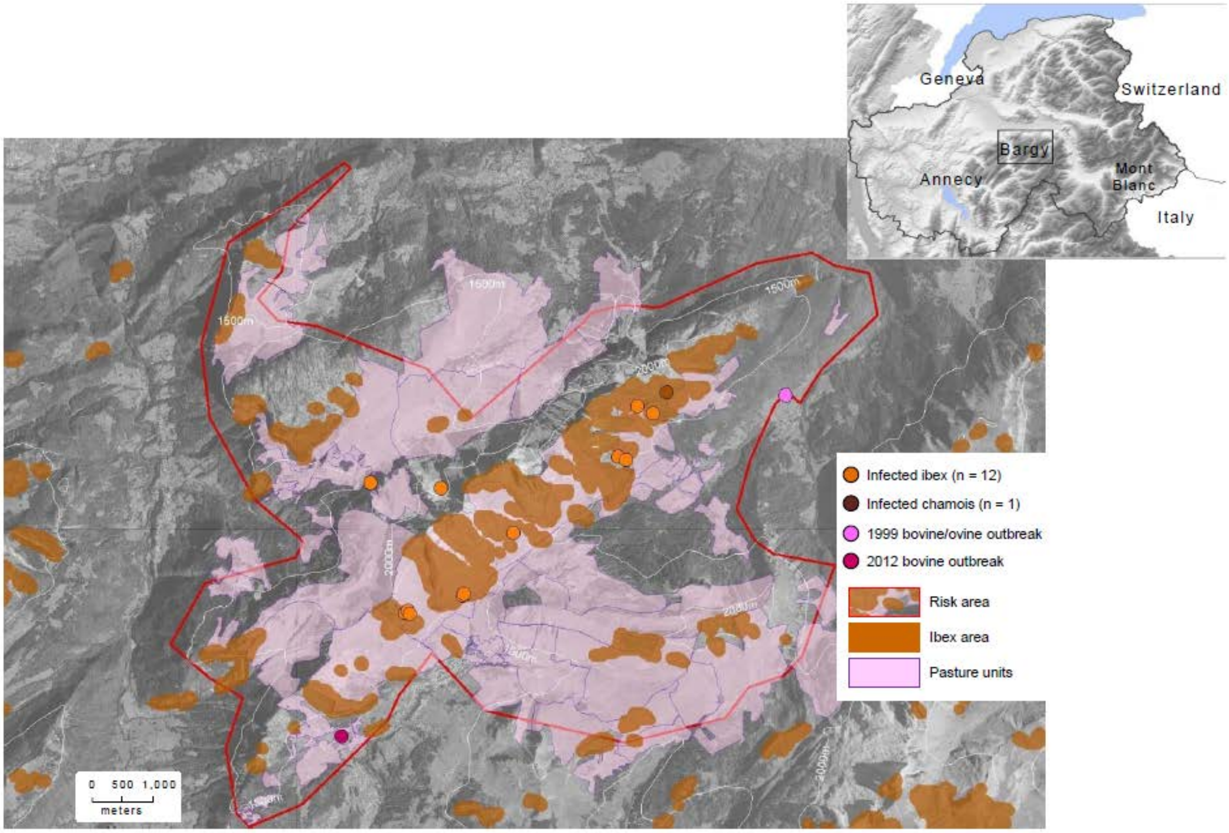

Technical Appendix Figure. Areas in France in which alpine ibex and chamois were found infected with *Brucella melitensis* during the study and in which brucellosis outbreaks occurred in ruminants during 1999 and 2012. The region shown is Bargy Massif, which is part of Bornes Massif in Haute-Savoie, France, and borders Switzerland and Italy. The elevation range for most of Bargy Massif is 900–2,300 m. However, the highest point has an elevation of 2,438 m. Alpine ibex areas were defined according to 3 criteria: >20% slope; region (southern, southwestern, and southeastern); and rocky substratum. The risk corresponds to the area in which the probability of cohabitation of wild and domestic ruminants is high.
